# Curcumin supplementation combined with high intensity interval training modulates serum irisin and lipid profile in obese women: "A randomized double-blind clinical trial"

**DOI:** 10.1016/j.conctc.2025.101464

**Published:** 2025-02-21

**Authors:** Bahram Jamali Gharakhanlou, Solmaz Babaei Bonab, Akram Amaghani, Mohammad Reza Shiri-Shahsavar

**Affiliations:** a Nutrition Research Center, Tabriz University of Medical Sciences, Tabriz, Iran; b Department of Sports Sciences, Faculty of Humanities, University of Maragheh, Maragheh, Iran; c Metabolic Diseases Research Center, Research Institute for Prevention of Non-Communicable Diseases, Qazvin University of Medical Sciences, Qazvin, Iran; d Clinical Trial Research Center, School of Medicine, University College Dublin, Dublin, Ireland

**Keywords:** Curcumin nanomicelle, High intensity interval training, Irisin, Lipid profile

## Abstract

**Background:**

Curcumin is the most widely known active substance of turmeric extract, which attributed numerous functional properties, including anti-inflammatory and cardioprotective properties. Inactivity and changes in lifestyle and subsequent overweight/obesity are becoming major health risk factors.

**Objective:**

the aim of this study was to determine the effect of curcumin supplementation combined with high intensity interval trainings (HIIT) exercise on serum irisin and lipid profile in obese women.

**Methods:**

40 inactive women (aged 30–35 years, and body mass index 30 and above) allocated in four groups (10 in each) as followed; curcumin (C), exercise plus curcumin (EC), placebo (P), and exercise plus placebo (EP). All subjects completed an eight-week HIIT program. The C and EC groups received one capsule containing 80 mg curcumin nanomicelle daily throughout study. Blood samples were taken in the beginning and after eight weeks to measure changes in the study variables (irisin and lipid profile). The ANCOVA and Bonferroni's post hoc test was used to compare variables between 4 groups and pairwise at a significance level of ≤0.05 using SPSS-22.

**Results:**

The results revealed that mean serum irisin (p = 0.023), cholesterol (p = 0.019), triglyceride (p = 0.022), high-density lipoprotein (p = 0.009) and low-density lipoprotein (p = 0.011) were significantly changed in all intervention groups compared to the placebo group.

**Conclusion:**

Based on these results, it can be concluded that HIIT training with curcumin consumption has a greater significancy on obesity reduction in women; Therefore, curcumin and HIIT exercise can be considered as a therapeutic approach to reduce the negative outcomes of obesity.

**Clinical trial registry number:**

IRCT20141004019397N2, link.

**Statement of significance:**

This study investigated the effects of curcumin supplementation combined with high-intensity interval training (HIIT) on human serum Irisin levels for the first time, in a clinical randomized trial. This suggests that HIIT training alongside curcumin consumption may be a promising therapeutic approach to reduce the negative health consequences of obesity in women.

## Highlights

• Curcumin and HIIT exercise may offer a therapeutic approach to mitigate the negative consequences of obesity.
• Curcumin + HIIT significantly improved irisin levels and lipid profiles in obese women.
• This combination holds promise as a therapeutic approach for combating obesity.

## 1 Introduction

Inactivity and changes in lifestyle and subsequent overweight/obesity are becoming major health risk factors in human lifespan. The prevalence of obesity in many countries, including Iran, is highly dependent on gender, age, and socioeconomic factors. In addition, marital status, physical activity level, urbanization, and type of diet are the main variables that are related to overweight and obesity in adulthood [1]. A wide range of behavioral, genetic, biological, and environmental factors are involved in the development of obesity, however, energy imbalance caused by physical inactivity, and slightly higher energy intake, is considered the most implementing factor in obesity [2]. Nevertheless, the molecular/cellular mechanism in adipose tissue is still not well studied. Clearly, exercise decrease the relative amount of adipose tissue in the muscles [3]. Recent studies reported that physical activity stimulates the release of a hormone called “irisin” from muscle tissue, which reduces body fat mass in several ways [4]. Irisin is a 211-amino-acid myokine that was recently introduced by Bostrom and colleagues in 2012 [5]. It is kind of a signaling protein that is released by skeletal muscles after the proteolysis of the membrane protein FNDC5, probably through contact with unknown receptors in white or gray adipose tissue. Irisin acts as an energy-consumption stimulating signal by increasing the expression of peroxisome proliferated activated receptors (PPARs) and is directly related to reduction in white adipose tissue [5] which is crucial for human metabolic diseases amendment. Metabolic disorders that are improved with exercise, attributed to be mediated by irisin signaling due to its role in mitochondrial biogenesis and oxidative metabolism in many cells [3]. Increment in total energy consumption in muscles, reduces body weight and insulin resistance in obese people [4]. In fact, irisin has a significant negative relationship with insulin resistance and obesity [5].

The use of synthetic drugs that are most widely prescribed can have serious side effects. Medicinal plants possess antioxidant properties due to the presence of special components, including polyphenols. In recent years, they have garnered attention as a potential therapeutic approach that may have no adverse effects [6]. The turmeric plant is home to one of these components. Among its most significant therapeutic effects are its antioxidant, anti-inflammatory, and anti-cancer properties, all of which can be attributed to curcumin, its most crucial active substance [7]. Additionally, curcumin possesses remarkable functional properties such as lowering cholesterol levels and preventing cardiovascular diseases [6]. Based on our literature review, no specific side effects and toxicity of curcumin (up to 8 g per day) have been reported in humans [8,9].

Regular physical activity, particularly moderate to high intensity exercises for three sessions per week, is widely regarded as an effective approach in the prevention and treatment of overweight and obesity across various age groups [10]. High intensity interval training (HIIT) has emerged as a prominent sports protocol in recent years. This form of exercise has gained significant interest from both experts and the general population owing to its appealing nature, diverse range of activities, and metabolic adaptability when compared to conventional endurance training. HIIT incorporates active and timed recovery periods despite traditional endurance training, which involves repetitive short-term activities with maximum intensity. This unique combination of intense bursts of exercise followed by brief recovery intervals has contributed to its popularity [11]. According to recent research findings, HIIT has been identified as a potent stimulus for enhancing various factors, such as irisin [12]. As an instance, HIIT exercise was more efficient in weight loss than traditional long-term endurance exercises [13]. In another study on obese men, Tsirigkakis et al. (2021) demonstrated that HIIT exercise improve body composition and increase fat oxidation [14]. Jafari et al. (2019) and Tofighi et al. (2017) studied young obese men and inactive obese women, respectively, and showed that after 8 weeks of HIIT exercise, the serum level of irisin increased significantly compared to the control group [15,16]. While in Eaton et al.'s study (2018), irisin concentration did not changed significantly after a 20-days period of HIIT exercise in healthy men [17]. However, in a study conducted by Vahdat et al. (2018), after six weeks of HIIT intervention in obese men, no significant increase in serum irisin was observe between groups [18].

Numerous conflicting findings have emerged regarding the impact of different physical activities, particularly HIIT protocol, on plasma irisin and lipid profile. Based on literature review, the combined effect of HIIT exercise and curcumin simultaneously has not been examined in human clinical trials. Therefore, the aim of this research was to explore the interactive influence of HIIT exercise and curcumin consumption on plasma irisin levels and lipid profile in sedentary obese women.

## 2 Materials and methods

### 2.1 Study design

**Fig. 1:**
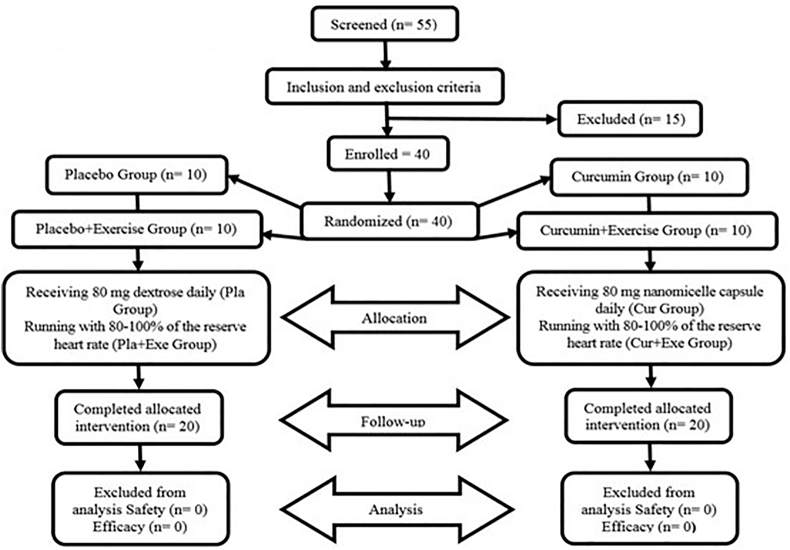
Study flowchart.

The current research was a double-blind clinical trial with a pre-test post-test design. The statistical population were obese women aged 30–35 years, and body mass index 30 and above who were engaged in sports clubs in last week were voluntarily included in the study. Individuals who were using drugs, supplements and alcoholic beverages, as well as smokers were excluded from the study. Additionally, those with a medical history, cardiovascular issues, high blood pressure, musculoskeletal and metabolic diseases limiting physical activity, allergies to spices and unwillingness to cooperate were also excluded. 55 obese women were volunteered to participate in our study, and after excluding 15 individuals, 40 individuals assigned to the study groups using random blocks with a volume of 4 generated by STATA14 software. The random assignment ensured that the groups were matched for age and Body mass index (BMI). The subjects were divided into 4 groups of 10 as follows: curcumin (C), exercise plus curcumin (EC), placebo (P), and exercise plus placebo (EP). Before initiation, the exercise protocol, the correct way of performing the exercises and possible risks were explained in a briefing session, and all participants filled the informed consent and physical health form. Throughout the intervention period and 48 h before each test, the subjects were instructed to maintain their usual behavior, eating habits, and refrain from consuming foods containing curcumin (such as turmeric, ginger, and cardamom) (Fig. 1).

### 2.2 Measurements

Anthropometric variables were assessed by taking weight measurements with minimal clothing and without shoes, utilizing a Sega digital scale with a precision of 100 g. Height was measured using an inflexible tape measure while the individuals stood next to a wall. Additionally, the BMI was calculated by dividing the weight in kilograms by the square of the height in meters [19]. The body fat percentage was determined using the Yagami Caliper, Mikusha model (manufactured in Japan), and the three-point formula recommended by the American College of Sports Medicine. This formula involved measuring three skin folds, namely the triceps, suprailiac, and thigh folds, and applying the Jackson formula - Pollock method (Supplementary 1), [20].

### 2.3 Physical activity

The physical activity levels of all participants were assessed at the commencement and conclusion of the intervention through the utilization of the IPAQ-SF questionnaire, which is the Persian version. This questionnaire has been proven to be valid and reliable and has been widely employed in domestic research studies [21]. The total physical activity is determined by multiplying the metabolic equivalent level (MET) for each physical activity by the duration of its performance per week. The resulting MET-minutes/weeks are then added together to obtain the overall measure of physical activity.

### 2.4 Nutritional information

All individuals were given the necessary training to use home scales and food albums to record a 24h recall for two normal days and one holiday at the beginning and end of the study. Nutritionist IV software was used to food analysis, and the average daily consumption of energy and macronutrients in daily energy supply was determined.

### 2.5 Curcumin supplementation

Participants in the curcumin groups received a single, daily dose of one licensed 80 mg curcumin nanomicelle capsule between 9 a.m. and 12 noon (produced by Mino Pharmaceutical Co.), while subjects in the placebo group took dextrose capsules at the same time. The content of capsules was not disclosed to either the participants or the researcher.

### 2.6 Exercise protocol

The training protocol was implemented 4 times a week, and 8 weeks inside the gym with suitable temperature and humidity. In the EP group, intense intermittent exercises were employed in the form of running for 20 s followed by periods of rest through walking, as outlined in Table 1. The intensity considered to be 80–100 % of the reserve heart rate according to the Karvonen method [22]. Prior to the main activity, a warm-up routine of 15 min consisting of soft jogging and stretching was performed. Following the main activity, a cooling down for 10 min was applied which included stretching and walking [23]. Subsequently, the heart rate was controlled by the researcher using a polar heart rate monitor until the end of the protocol.

**Table 1:** HIIT protocol in interventional groups The intensity of training, repetition and number of sets during 8 weeks of study.

Weeks	Training to Rest Ratio (seconds)	Intensity	Repetitions	Sets	Rest between sets	Sessions per week	Total Time (minutes)
1	20:160	80–90 % (Reserve Heart Rate)	4	3	5 Minutes (Inactive)	4	46^a^
2	20:150						44
3	20:140						57
4	20:130			4			55
5	20:120	90–100 % (Reserve Heart Rate)					66
6	20:110						63
7	20:110			5			68
8	20:100						65

a 20 s of activity followed by 160 s of rest between repetitions equals 3 min With 4 repetitions, this totals 12 min. Three sets, each lasting 12 min, comes to 36 minutes. Finally, adding two 5-min rest periods between the three sets, the total duration is 46 min.

### 2.7 Blood samples preparation and laboratory measurements

Blood samples were collected 24h prior to the commencement of the experimental procedure and after the completion of the final training session. At each stage, a volume of 4.5 ml was extracted from the antecubital vein in left arm of the participants. Samples were left for few minutes to coagulate and serum were separated after centrifuge and eventually refrigerated in −70 °C till the biochemical assessments. Measurements were conducted between 9 and 12 in the morning, and under the same standard conditions for all groups. To assess irisin, the ELISA method was used according to the instructions of the kits (Cusabio, JPN). Lipid profile (total cholesterol (TC), triglyceride (TG) and high-density lipoprotein (HDL)) investigated using Pars kits and by photometric method. Friedwald's formula was used to calculate low-density lipoprotein (LDL) [19].

### 2.8 Statistical analysis

The normality of data distribution was assessed using the Shapiro-Wilk test. To compare qualitative variables between groups, either the Chi-Square or Fishers exact test was employed. For the comparison of quantitative variables between study groups, the One-Way ANOVA test was utilized. Additionally, the paired *t*-test was employed to compare quantitative variables before and after the intervention within each group. To compare quantitative variables among the four groups, the ANCOVA test was conducted with adjustment for baseline values. Pairwise comparisons between the four groups were performed using the Bonferroni post hoc test. All statistical operations and analyses were carried out using SPSS version 22 statistical software, with a significance level set at five percent (cc ≤ 0.05).

### 2.9 Ethical considerations

The current study was approved by the Research Ethics Committee of Tabriz University of Medical Sciences, with assigned No. IR.TBZMED.REC.1400.314. Additionally, it was duly registered in the Clinical Studies of Iran, bearing the code IRCT20141004019397N2. The confidentiality of all information gathered from the participants was strictly maintained, ensuring their privacy and anonymity. Moreover, the participants had the freedom to withdraw from the study at any point without any consequences. Prior to the commencement of the study, the participants provided their informed consent by signing a written document, and the subjects were provided with comprehensive explanations regarding the research objectives and the methodology employed. If any queries arose during or after the study, they were properly answered by the project manager.

## 3 Results

The average physiological and anthropometric characteristics of the subjects in the pre-test stage, categorized according to the study groups in Table 2. The findings indicate that all the physiological and anthropometric indicators, along with food intake, follow a normal distribution. No significant differences were observed among the four studied groups at the baseline age (P-value = 0.23), BMI (P-value = 0.35), body fat percent (P-value = 0.32), systolic blood pressure (P-value = 0.48), diastolic blood pressure (P-value = 0.29), and physical activity (P-value = 0.23).

**Table 2:** Physiological characteristics of subjects in different groups.

Variable	Groups				P- value
	P (n = 10)	C (n = 10)	EP (n = 10)	EC (n = 10)	
Age (year)	33.11 ± 1.7	32.09 ± 2.4	32.1 ± 1.8	33.4 ± 1.5	0.23
BMI (kg/m2)	32.8 ± 1.58	32.7 ± 2.22	31.85 ± 1.58	34.7 ± 3.41	0.35
Body fat percentage (%)	33.76 ± 3.7	33.66 ± 3.85	35.76 ± 4.64	34.8 ± 3.41	0.32
Systolic blood pressure (mm Hg)	121 ± 12	125 ± 6	119 ± 8	122 ± 11	0.48
Diastolic blood pressure (mm Hg)	83 ± 4	84 ± 5	79 ± 7	78 ± 9	0.29
physical activity (MET-min/WK)	501.2 ± 122.5	491.8 ± 150.7	475.3 ± 195.4	365.6 ± 214.2	0.23

Values are reported as mean ± standard deviation. curcumin (C), exercise plus curcumin (EC), placebo (P), exercise plus placebo (EP).

We utilized 24-h recalls to control dietary confounding factors, and for monitoring intervention-related effects on nutrient intake and avoiding possible bias in the study outcomes. Mean energy and nutrient intake in the study groups are presented in Table 3. No significant differences were observed among the four studied groups (P-value >0.05).

**Table 3:** Mean energy and nutrient intake of the groups before and after intervention.

Variable		Groups			
		P (n = 10)	C (n = 10)	EP (n = 10)	EC (n = 10)
Energy (Kcal/day)	Pre- test	1768.3 ± 340.2	1685.5 ± 205.7	1850.3 ± 326.1	1966.1 ± 301.2
	Post- test	1743.6 ± 355.4	1654.3 ± 309.1	1862.6 ± 196.4	1915.3 ± 326.1
	**P-value**	0.513	0.426	0.759	0.286
Carbohydrate (g/d)	Pre- test	236.5 ± 57.3	218.7 ± 35.5	219.1 ± 54.3	246.4 ± 45.3
	Post- test	238.8 ± 89.6	241.3 ± 74.4	255.8 ± 35.2	258.2 ± 55.4
	**P-value**	0.861	0.334	0.125	0.532
Protein (g/d)	Pre- test	53.6 ± 34.3	54.8 ± 15.6	57.3 ± 18.5	67.7 ± 13.1
	Post- test	52.1 ± 8.6	53.3 ± 11.2	58.2 ± 11.8	62.3 ± 10.2
	**P-value**	0.541	0.512	0.795	0.071
Total fat (g/d)	Pre- test	59.5 ± 19.3	58.3 ± 19.1	71.5 ± 22.9	68.4 ± 16.3
	Post- test	58.8 ± 25.4	48.2 ± 21.9	58.8 ± 11.6	63.9 ± 11.8
	**P-value**	0.931	0.245	0.068	0.256
Zinc (mg/d)	Pre- test	5.35 ± 1.67	4.97 ± 2.54	5.54 ± 3.22	6.79 ± 2.91
	Post- test	4.56 ± 2.14	3.89 ± 1.18	5.37 ± 1.46	5.58 ± 2.21
	**P-value**	0.149	0.262	0.864	0.232
Selenium (mg/d)	Pre- test	0.04 ± 0.01	0.04 ± 0.03	0.05 ± 0.03	0.08 ± 0.06
	Post- test	0.09 ± 0.10	0.06 ± 0.03	0.09 ± 0.06	0.13 ± 0.08
	**P-value**	0.156	0.222	0.054	0.084
Vit E (mg/d)	Pre- test	4.56 ± 5.92	2.52 ± 1.34	4.15 ± 6.32	3.52 ± 4.85
	Post- test	3.89 ± 2.56	3.09 ± 3.22	2.89 ± 2.05	3.45 ± 2.31
	**P-value**	0.36	0.07	0.63	0.91
Vit C (mg/d)	Pre- test	77.6 ± 39.4	107.5 ± 102.1	90.6 ± 65.3	87.5 ± 67.3
	Post- test	111.9 ± 186.2	129.4 ± 182.3	71.2 ± 46.2	64.1 ± 44.1
	**P-value**	0.64	0.79	0.42	0.38

Values are reported as mean ± standard deviation. curcumin (C), exercise plus curcumin (EC), placebo (P), exercise plus placebo (EP).

**Fig. 2:**
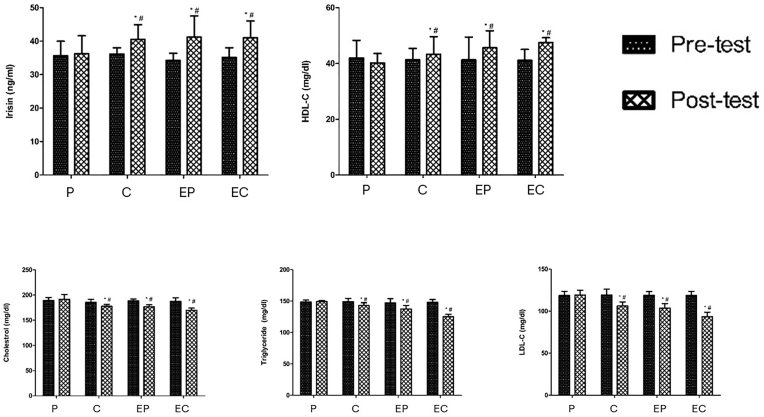
Mean changes of irisin and lipid profile in curcumin (C), exercise plus curcumin (EC), placebo (P), exercise plus placebo (EP) groups before and after intervention. Values are reported as mean ± standard deviation. ∗ Significancy compared to pretest values (P > 0.05), # Significancy compared to placebo (P > 0.05).

The results indicate that there were significant changes in serum irisin and lipid profile levels among the studied groups from the pre-test to the post-test (Fig. 2, Table 4). One-way ANOVA test results for the pre-test indicated no significant difference in mean serum irisin and lipid profile (TC, TG, LDL and HDL) levels among the studied groups all (P-values >0.05, all P-values and related test results are presented in Table 4). However, there was a significant difference among the groups in the post-test (P-values ≤0.05). Paired *t*-test analysis in post-test demonstrated a significant increase in serum irisin and HDL levels in curcumin, EC and EP groups (P-values ≤0.05). In addition, a significant decrease in lipid profile was observed in the post-test compared to the pre-test (P-values ≤0.05) in intervention groups.

**Table 4:** Mean changes of irisin and lipid profile in curcumin (C), exercise plus curcumin (EC), placebo (P), exercise plus placebo (EP) groups before and after intervention. Values are reported as mean ± standard deviation. Significance was set at p ≤ 0.05. a One-way ANOVA test, b Paired sample t-test, c ANCOVA test adjusted for baseline values (for among-group comparisons at the end of the study), d Bonferroni test adjusted for baseline values and compared to the placebo group.

Variable (Units)	EC	EP	C	P	P-value	P-value EC/P,^d^	P-value EP/P,^d^	P-value C/P,^d^
**Irisin (ng/ml)**								
Pre-test	35.2 ± 15.88	34.2 ± 25.15	36.1 ± 14.87	35.4 ± 6.36	0.198^a^	–	–	–
Post-test	41.5 ± 1.03	41.6 ± 21.31	40.4 ± 48.44	36.5 ± 21.4	0.042^a^	–	–	–
P-value^b^	0.001	0.001	0.049	0.238	0.012^c^	0.004	0.011	0.047
**Cholesterol (mg/dl)**								
Pre-test	187.7 ± 34.2	188.3 ± 25.9	185.5 ± 34.98	189.5 ± 15.8	0.329^a^	–	–	–
Post-test	169.4 ± 45.7	176.4 ± 43.1	177.3 ± 45.9	191.9 ± 5.4	0.033^a^	–	–	–
P-value^b^	0.001	0.021	0.039	0.325	0.009^c^	0.007	0.038	0.043
**Triglycerides (mg/dl)**								
Pre-test	148.5 ± 14.4	147.6 ± 17.8	149.5 ± 12.2	148.3 ± 45.3	0.563^a^	–	–	–
Post-test	125.3 ± 23.7	137.6 ± 47.5	143.4 ± 16.3	149.1 ± 23.8	0.002^a^	–	–	–
P-value^b^	0.001	0.001	0.019	0.541	0.011^c^	0.001	0.017	0.025
**LDL (mg/dl)**								
Pre-test	118.65 ± 4.9	118.87 ± 4.4	119.12 ± 1.7	118.65 ± 4.9	0.487^a^	–	–	–
Post-test	93.35 ± 5.4	103.53 ± 5.5	106.17 ± 4.8	119.23 ± 5.6	0.017^a^	–	–	–
P-value^b^	0.001	0.024	0.041	0.402	0.008^c^	0.001	0.011	0.043
**HDL (mg/dl)**								
Pre-test	41.3 ± 12.9	41.8 ± 28.18	41.4 ± 31.1	41.4 ± 98.3	0.583^a^	–	–	–
Post-test	47.1 ± 53.1	45.5 ± 61.12	43.6 ± 33.3	40.3 ± 17.4	0.001^a^	–	–	–
P-value^b^	0.005	0.018	0.047	0.397	0.001^c^	0.001	0.001	0.018

ANCOVA test, which was performed by adjusting the effects of baseline values, demonstrated a significant difference in the irisin and lipid profile between the studied groups at the end of intervention (P-values ≤0.05). Moreover, results of Bonferroni's post hoc analysis, which was performed by adjusting the baseline values, showed that the serum irisin and HDL increased significantly in all intervention groups compared to the placebo group; However, there was a significant decrease in TC, TG and LDL in all groups compared to the placebo group (P-values ≤0.05) (Fig. 2).

## 4 Discussion

This study investigated the effect of 8 weeks of curcumin supplementation and HIIT exercise on irisin levels and lipid profile in inactive obese women. The results showed that 8 weeks of HIIT exercise and curcumin intake led to an increase in irisin and improvement of lipid profile in the intervention groups compared to the placebo group. Previous research has demonstrated that engaging in physical exercise boosts irisin secretion, with its release rate influenced by factors like exercise type, duration, and intensity [4]. This can result in heightened irisin levels both during and immediately following physical activity [24]. Bostrom et al. (2012) reported that irisin gene expression depends on the type and duration of training, while in their research, 3 weeks of running in rats and 10 weeks of endurance training in healthy adults had different effects on irisin levels. Endurance training is likely to modulate the availability of fuel sources during exercise, creating an energy deficit that triggers metabolic pathways involved in regulating the expression of the irisin gene, consequently boosting irisin secretion [5]. Moreover, the intensity of training is crucial for maximizing the impact of the training on irisin levels. Pekkala et al. (2013) compared the irisin expression levels induced by intense aerobic exercise and moderate intensity combined exercise. Serum irisin increased immediately after either exercise protocols in their study, with a more pronounced increase observed after intense aerobic exercise. This suggests that exercise intensity influences irisin expression [4]. In another study by Jafari et al. (2019), intense interval training for eight weeks (three/week) led to a significant increase in serum irisin levels in young obese men [15]. In Tofighi et al.'s study (2017), eight weeks of intense interval training significantly increases irisin serum levels in inactive obese women [16]. These results are in accordance with our study, while Eaton et al.’s (2018) research is in contradiction with the findings of the current research. They reported that irisin concentration did not change significantly after 20-days of HIIT training in healthy men [17]. In addition, Hecksteden et al. (2013) conducted a 26-week aerobic training intervention at 60 % heart rate in young individuals. They found no significant difference in irisin levels between the training and control groups. The authors attributed this lack of effect to the prolonged freezing of serum samples, which may have degraded irisin and compromised their results [25]. The variations in outcomes concerning the impact of physical exercise and curcumin supplementation on irisin may result from individual differences, genetic and dietary factors, as well as variations in exercise protocols, intensity, duration, participant physical condition, body weight, age, gender, muscle mass, brown fat tissue, body mass index, and differences in serum sample storage time and measurement methods across studies.

Based on our literature review, high-intensity interval training is more effective for weight loss than prolonged endurance training and can help prevent the effects and illnesses associated with obesity [13]. Furthermore, skeletal muscle acts as an endocrine organ capable of releasing various signaling molecules and regulatory cytokines known as myokines, such as irisin, which plays a role in controlling numerous physiological and pathological processes. It appears that the rise in irisin levels following vigorous intermittent exercise may be attributed to peroxisomal proliferator-activated receptor-γ coactivator-1α (PGC-1α) activation signals. Physical activity can boost FNDC5 in muscle tissue by enhancing PGC-1α. FNDC5 is a membrane protein that secretes the myokine irisin after proteolytic degradation [26]. Conversely, Timmons et al. (2012) did not detect an association between PGC-1α and FNDC5. It appears that the genes influenced by exercise depend on the type of activity, and regulating FNDC5 gene expression alone may not fully demonstrate exercise's health benefits. Moreover, the researchers demonstrated that alterations in FNDC5 gene expression did not align consistently with variations in serum irisin levels, suggesting that additional factors may play a role in irisin release from muscle [27]. Irisin upregulates UCP1 gene expression and other genes linked to brown fat tissue by enhancing PPAR-γ expression through binding to unidentified receptors in white fat cells and other tissues. This upregulation of UCP1 results in the dissipation of energy as heat, leading to heightened energy expenditure. Consequently, irisin serves as an energy utilization signal that enhances metabolic function, and through elevating overall energy expenditure, may contribute to weight loss [28]. Additionally, prior studies have explored the potential connection between diet and irisin secretion, revealing that dietary patterns do not alter irisin levels [29,30]. In general, physical activity promotes overall fat oxidation in adipose tissue, muscle tissue, and liver tissue, culminating in reduced circulating fatty acids.

In the current study, combining intense interval training with curcumin intake has culminated in significant reduction in cholesterol, triglycerides, and LDL levels, whilst a marked increase in HDL levels was observed. These findings align with several previous studies [23,31,32], but contradict the results reported by Kraus et al., in 2003 [33]. The decrease in plasma triglycerides may stem from heightened triglyceride breakdown facilitated by increased lipoprotein lipase (LPL) activity. El-Moselhy et al. (2011) highlighted the significant effect of curcumin intake on triglyceride levels [34]. Elevated LPL activity triggers the decomposition of triglycerides and lipid-rich lipoproteins, enhancing triglyceride removal from the bloodstream. These triglyceride alterations can manifest independently of changes in body composition. Several studies have demonstrated that curcumin inhibits fatty acid synthase activity and enhances fatty acid beta-oxidation, resulting in effective fat storage reduction [31].

The researchers have recently found that Curcumin alleviated obesity, reduced inflammation in white adipose tissue (WAT), and improved insulin sensitivity in mice feeding high fat diet [35]. This study investigates its beneficial metabolic effects in animal settings. These effects were associated with increased plasma irisin levels, enhanced metabolic activity in brown fat and inguinal WAT, and improved basal metabolic rate. They suggested that curcumin's positive impact on energy metabolism was linked to the activation of the FNDC5/p38 MAPK/ERK pathway, which subsequently may improve metabolic health by regulating energy expenditure and promoting the release of irisin, a hormone linked to improved insulin sensitivity and energy metabolism [35]. Recent study investigates the effects of curcumin and 8 weeks aerobic exercise on hyperandrogen-induced endoplasmic reticulum (ER) stress and ovarian cell apoptosis in rats with polycystic ovary syndrome (PCOS) [36]. The study found that both curcumin and aerobic exercise effectively reduced ER stress and GC apoptosis in PCOS rats, improving ovarian function and follicular development. These effects were mediated by inhibiting the hyperandrogen-activated ER stress IRE1-α/XBP1 pathway, suggesting a potential therapeutic approach for PCOS treatment. Dihydrotestosterone induced ER stress was mitigated by curcumin/irisin in primary ovarian cell culture.

In conclusion, the current research indicates that combining intense interval training with curcumin consumption yields significant benefits for obese women. Consequently, it is suggested that obese women incorporate intense interval training alongside curcumin intake to mitigate the adverse impacts of obesity and manage their weight effectively.

This study investigated the effects of physical activity and curcumin on individuals with obesity, a significant health issue affecting a large population suffering from obesity/overweight, and their related complications. The strength of this study lies in its novel approach to combining curcumin supplementation with HIIT to explore their effects on serum irisin levels and lipid profiles in obese women and target the obesity complications. By employing a randomized controlled trial design, this research effectively minimized bias, enhancing the validity of the findings. The study defined its experimental groups clearly and focused on important health markers, such as irisin, cholesterol, triglycerides, HDL, and LDL levels. This research not only contributes to the growing body of literature on curcumin and exercise but also highlights practical strategies for addressing obesity-related health challenges, ultimately aiming to improve the well-being of individuals struggling with weight management. However, limitations such as the sample size, short age range, and only female participants remains to be discussed. Future studies are recommended to address these limitations and establish an optimal dose for curcumin.

## CRediT authorship contribution statement

**Bahram Jamali Gharakhanlou:** Writing – original draft, Resources, Funding acquisition, Conceptualization. **Solmaz Babaei Bonab:** Writing – original draft, Methodology, Investigation, Funding acquisition. **Akram Amaghani:** Visualization, Methodology, Investigation. **Mohammad Reza Shiri-Shahsavar:** Writing – review & editing, Conceptualization.

## Data availability

The datasets generated and/or analyzed during the current study are available from the corresponding author on reasonable request. In addition, all data generated or analyzed during this study are included in this published article.

## Author disclosures

The authors declare they have no conﬂict of interest.

## Funding

This study was funded by a grant from ‏the ‘Research Vice-Chancellor’ of 10.13039/501100007831Tabriz University of Medical Science, Tabriz, Iran.

## Declaration of competing interest

The authors declare that they have no known competing financial interests or personal relationships that could have appeared to influence the work reported in this paper.
