# Supplementary material for: Curcumin supplementation combined with high intensity interval training modulates serum irisin and lipid profile in obese women: "A randomized double-blind clinical trial"
Source: Contemp Clin Trials Commun. 2025 Feb 21;44:101464. doi: 10.1016/j.conctc.2025.101464 (PMC11909447; doi:10.1016/j.conctc.2025.101464)
Supplement: Multimedia component 1 [file mmc1.docx]

**Supplementary 1**

There are different variations of the formula based on gender and the number of skinfold sites measured. The Jackson-Pollock formula is used to estimate body fat percentage based on skinfold thickness measurements. It's important to take each measurement multiple times (2-3) and average them for accuracy. Here are the most common versions:

**For Men (3-Site Formula)**

1. **Skinfold Sites**: Chest, Abdomen, Thigh
2. **Formula**: Body Density=1.10938−0.0008267×Sum of Skinfolds+0.0000016×(Sum of Skinfolds)^2^
3. **Body Fat Percentage**: = ((4.95/Body Density)−4.50)/100

**For Women (3-Site Formula)**

1. **Skinfold Sites**: Triceps, Suprailiac, Thigh
2. **Formula**: Body Density=1.0994921−0.0009929×Sum of Skinfolds+0.0000023×(Sum of Skinfolds)^2^
3. **Body Fat Percentage = ((4.95/Body Density)−4.50)/100**
